# Socioeconomic inequities patterns of multi-morbidity in early adolescence

**DOI:** 10.1186/1475-9276-12-65

**Published:** 2013-08-20

**Authors:** Kénora Chau, Michèle Baumann, Nearkasen Chau

**Affiliations:** 1 Université de Lorraine, Vandoeuvre-lès-Nancy, France; 2 University of Luxembourg, INtegrative research unit on Social and Individual DEvelopment (INSIDE), Walferdange, Luxembourg; 3 INSERM, U669, Paris F-75014, France; 4 Univ Paris-Sud and Univ Paris Descartes, UMR-S0669, Paris, France; 5 Inserm, U669, 8 rue du Breuil, F-54180 Heillecourt, France

**Keywords:** Adolescents, Multi-morbidity, Mental health difficulties, Unhealthy behaviors, Drugs consumptions, Obesity, Socioeconomic factors, Social inequalities

## Abstract

**Background:**

Multi-morbidity such as cumulating mental health, behavioral, and school difficulties (consumptions of alcohol, tobacco, cannabis, and hard drugs, obesity, depressive symptoms, suicide attempts, involvement in violence, and low school performance) is common in early adolescence and can be favored by a number of socioeconomic factors (gender, age, nationality, family structure, parents’ education, father’ occupation, and income). This study assessed the concurrent roles of various socioeconomic factors in multi-morbidity defined as cumulated number of difficulties (CD) which has been partially documented.

**Methods:**

Adolescents from middle schools in north-eastern France (N = 1,559) completed a questionnaire measuring socioeconomic characteristics and mental health, behavioral, and school difficulties. Data were analyzed using logistic regression models.

**Results:**

Alcohol use affected 35.2% of subjects, tobacco use 11.2%, cannabis use 5.6%, hard drugs use 2.8%, obesity 10.6%, depressive symptoms 13.3%, suicide attempts 9.9%, involvement in violence 10.3%, and low school performance 8.2%. Insufficient income and non-intact families impacted most mental health, behavioral, and school difficulties with adjusted odds ratios (ORa) between 1.51 and 3.72. Being immigrant impacted illicit drugs use and low school performance (ORa 2.31-4.14); low parents’ education depressive symptoms (1.42) and school performance (3.32); and manual-worker/inactive offspring low school performance (2.56-3.05). Multi-morbidity was very common: CD0 44.1%, CD1 30.8%, CD2-3 18.4%, and CD ≥ 4 6.7%. Insufficient income, divorced/separated parents, reconstructed families, and single parents played impressive roles with strong ORa gradients (reaching 4.86) from CD1 to CD ≥ 4. Being European immigrant, low parents’ education, and low fathers’ occupations had significant gender-age-adjusted odds ratios for CD2-3 and CD ≥ 4, but these became non-significant when adjusted for all socioeconomic factors. Older adolescents had higher risks for multi-morbidity which did not change when adjusting for all socioeconomic factors.

**Conclusions:**

Multi-morbidity including a wide range of mental health, behavioral, and school difficulties was common in early adolescence. Insufficient income and non-intact families played impressive roles. Being immigrant, low parents’ education, and low fathers’ occupations also played strong roles but these were explained by insufficient income and non-intact families. Prevention against multi-morbidity should be designed to help adolescents to solve their difficulties, especially among adolescents with socioeconomic difficulties.

## Introduction

Youth development including healthy self-awareness and self-care behavior, future goal achievement, and successful transition into adulthood needs stable and nurturing contexts promoting achievement of trust, optimism, and meaning in life [1]. But many adolescents early suffer from a wide range of mental health and behavioral difficulties (substance uses, lack of sports/physical activity, obesity, depressive symptoms, suicide attempts, and involvement in violence) and school difficulties (grade repetition, low school performance, and school dropout) [2-9]. Multi-morbidity defined as cumulating several of mental health, behavioral, and school difficulties should be common because these share a number of potential factors. Indeed, many adolescents suffer from parents’ socioeconomic difficulties including low education, low social status, divorce/separation, death, and poverty [2-4] which are well known risk factors for mental health, behavioral and school difficulties [1,3-5,9-13]. These issues may be more common among immigrant adolescents as their families are originating from less developed or developing countries with lower gross domestic product per inhabitant [14], and have lower education, socioeconomic status, and resources [13,15,16].

Multi-morbidity may be caused by higher and more persistent socioeconomic vulnerabilities. Among socioeconomic factors, parents’ low education and low social status as social background may play important roles, but we may suspect that family social environment/functioning and poverty may play higher roles [17]. Indeed, one study reported that among middle school adolescents, parents’ divorce/separation and death early occur (median ages of occurring 6 and 8 years, respectively) [18] and they often result in poorer family environment and resources. According to the OECD [19] there are now more children living with cohabiting, divorced/separated or single parents. Over the past decade poverty in households with children is rising in nearly all OECD countries (12.7% across the OECD, and one in five children in Israel, Mexico, Turkey, the United States, and Poland) [19]. The previous study also reported that family issues were rapidly followed by mental health, behavioral and school difficulties: median age of occurring 10 years for depressive symptoms; 11 years for involvement in violence, suicide attempts, and alcohol use initiation; 12 years for tobacco use initiation; and 13 years for cannabis and hard drugs uses initiation [18]. Consequently, multi-morbidity early occurred among many adolescents.

Multi-morbidity may be an auto-amplified phenomenon i.e. a mental health, behavioral, or school difficulty may favor in turn other difficulties and so on. Indeed, involvement in violence is associated with developmental trajectory failure, depressive and internalizing symptoms, and suicide behaviors [1,2,6,8,20,21] through child maladaptation, stress physiology, damage to cognitive development, and psychopathology development [7,22,23]. School difficulty may progressively favor school disengagement, absenteeism, and psychological disorders [24]. Depression could alter executive functions, cognitive ability and work performance [25-27] leading to more living difficulties and drugs uses to cope [2,4,10-12,28-30]. Drugs uses, mostly initiated in adolescence may follow gateway patterns and escalation trajectories from alcohol and tobacco to cannabis and then to hard drugs [11,31-33]. Because their consumptions could alter physical and mental capacities in the short-term [34-37] they could increase in turn living difficulties, school and mental difficulties [36], leading the subjects to intensify their consumptions to cope, and so on. This risk patterns may partly explain escalade trajectories for certain adolescents [32,33] but also common multi-morbidity. The knowledge of the mechanisms through which various socioeconomic factors may impact multi-morbidity is critical to inform preventive interventions to limit it, especially among most vulnerable adolescents. However they have remained unclear as most studies have investigated few socioeconomic factors and rarely multi-morbidity [12,32,33,38]. According to our knowledge there has been no study on multi-morbidity among adolescents although it may be common because of high prevalence of mental health, behavioral, and school difficulties in various countries [1,2,5-8,39]. Studies on multi-morbidity have mostly concerned chronic diseases among adults and elderly people, which have pointed especially out the role of socioeconomic deprivation, family structure, income, need to receive continuity of care, and vigilance of health care professionals working across all medical specialties [40-47]. One study reported that the prevalence of multi-morbidity, defined as two or more diseases at the same time, was 10% in the 0–19 year age group and increased to 78% in subjects aged 80 years or over [48]. One study in Spain found that multi-morbidity affected all age groups and its prevalence increased from 13% in 15–44 year age group to 67% in subjected aged 65 years or over; it identified five patterns of multi-morbidity which were cardio-metabolic, psychiatric-substance abuse, mechanical-obesity-thyroidal, psychogeriatric, and depressive [49]. The World Health Organization underlines the need to improve the health and well-being of populations, reduce health inequalities, and ensure sustainable people-centred health systems [50].

This study assessed the associations between most potential socioeconomic factors (gender, age, being immigrant, family structure, parents’ education, father’ occupation, and income) with multi-morbidity including a large number of mental health, behavioral and school difficulties (consumptions of alcohol, tobacco, cannabis, and hard drugs, obesity, depressive symptoms, suicide attempts, involvement violence, and low school performance) in early adolescence in France. We focused on individuals in middle school students mostly under 16 years because school is compulsory until 16 years and many problems such as substance uses become persistent in late adolescence period (16–20 years) [12,32,33]. Thus mental health, behavioral and school difficulties and their risk factors should be identified, treated and monitored in early adolescence. Contrarily to other national studies we have participated [11,34,35] we chose to focus the present survey on the exhaustive population from a north-eastern French urban area so that the subjects are in the same socioeconomic context, free of variations across regions. Its health and health-related behaviors were close to those of the whole France.

## Methods

### Procedure

The study population comprised all 1,666 students attending three middle schools in a geographical area of the Nancy urban area (410,000 inhabitants) in north-eastern France. The population studied included all students from all the middle schools (two public and one private) in this area which included 63 classes. The study protocol included: an application to participate transmitted to parents/guardians via the students (April 2010), and data collection undertaken (May-June 2010) using an anonymous self-administered questionnaire in the course of a 1 h-class period, under the research team supervision with the help of teachers (when they wished, for surveillance with no influence on the survey). The completed questionnaire was put in a sealed envelope and then in a closed box by the subject. All were made to guarantee the respondents’ anonymity. For this purpose, the questionnaire excluded the birthday, the birth place, and the residential town. The investigation was approved by the Nancy-Metz regional education authority and the *Commission Nationale de l’Informatique et des Libertés* (national review board). Written informed consent was obtained from the respondents. Among the 1,666 subjects included in the population studied, 2 refused and 89 (5.3%) were absent when the data collection was carried out (for motive independent of the survey). In total 1,575 (94.5%) completed the questionnaire, of which 10 were of unknown gender or age, 9 were not completed appropriately, leaving 1,559 questionnaires for statistical analysis. The health and health-related behaviors of the sample were close to those of the whole France [2,51] (Table 1).

**Table 1:** **Comparison between the study sample and France (ESPAD survey **[2,51]** (%)**

	**Study population**	**France**
	**(limited to <16 years **^**a)**^**)**	**<16 years**
	**(N = 1,524)**	**(N = 8,367)**
Boys	49.9	48.9
Family structure		
Intact	63.2	74.7
Reconstructed	15.0	11.3
Single parent	16.4	11.7
Others	5.4	2.3
Last-30-day substance use		
Tobacco	10.7	13.6
Alcohol	34.7	34.6
Cannabis	5.1	5.5
Sleep disorders	32.6	29.0
Asthma	17.2	16.3
Depressive symptoms	13.1	9.8
Last-12-month suicidal ideation	11.6	9.1
Lifetime suicide attempts	9.6	7.2
Sexual abuse	3.4	1.9
Sustained physical/verbal violence (at least once)	53.3	51.5

^a)^ were excluded 35 subjects aged 16 years or over.

### Measures

The questionnaire included socioeconomic characteristics (gender, birth month and year, nationality, family structure, parents’ education, father’ occupation, and income) and mental health, behavioral and school difficulties (consumptions of alcohol, tobacco, cannabis, and hard drugs, obesity, depressive symptoms, suicide attempts, involvement in violence, grade repetition, and low school performance).

Five father’s occupational categories were considered following the international classification of occupation (ISCO): professionals, managers, and intermediate professionals (reference category); craftsmen/tradesmen/firm heads; service workers/clerks; manual workers and other occupations; and inactive people (unemployed and retirees) [4,25,52]. For perceived income, subjects were asked whether the financial situation of their family was: coping but with difficulties/getting into debt vs. comfortable/well off/earning just enough [25,52].

Last-30-day use of alcohol, tobacco, cannabis, and hard drugs were respectively assessed with the questions [2,12,51]: ‘During the last 30 days how many times have you had alcohol drinks (beer, cider, champagne, wine, aperitif, …?’ (none/1-5/6-9/10-29/30+), ‘During the last 30 days did you smoke cigarettes?’ (none/1-4/5-9/10-19/20+ cigarettes/day), ‘During the last 30 days how many occasions have you used any form of cannabis?’ (none/1-5/6-9/10-29/30+), and ‘During the last 30 days how many occasions have you used any form of other illicit drugs (mushrooms, ecstasy, LSD, …)?’ (none/1-5/6-9/10-29/30+). These factors were dichotomized (at least once vs. none).

Obesity was assessed using measured body height and weight. During questionnaire completion, adolescents were invited to measure their body weight and height with the same research-team trained physician. Body height was measured with a bodymeter measuring tape (mounted on a portable stadiometer fixed on the wall). Weight was measured with a Scaleman electronic balance (accuracy of 50 grams). Measurements were done without shoes in a light gown. Body mass index (BMI) was defined as weight/height^2^ (kg/m^2^). Obesity was defined according to the widely used threshold values recommended for male and female French adolescents at different ages [53].

Depressive symptoms were measured with the Kandel scale [2,54]. The Cronbach's alpha was satisfactory (0.84) allowing a single score to be calculated (range 6–18). They were defined by a score above the 90^th^ percentile value (≥17). Suicide attempts were addressed in the question ‘During the life course, how many times did you actually attempt suicide?’ (Any/None) [2].

Involvement in violence was measured with a 11-item scale: ‘During the last 12 months, have you?’: ‘gotten mixed into a fight in school’, ‘taken part in a fight where a group of your friends were against another group’, ‘belonged to a group starting a fight against another group’, ‘been author of verbal violence’, ‘been author of racial actions’, ‘started a fight with another individual’, ‘taken something not belonging to you (in school, in the neighborhood of school, at home, …’, ‘taken something from a shop without paying for it’, ‘set fire to somebody else's property on purpose’, ‘used any kind of weapon to get something from a person’, or ‘damaged public or private property on purpose’ (Any/None) [2]. The Cronbach's alpha was satisfactory (0.82), allowing a single score to be calculated as the number of positive responses. Involvement-in-violence was defined by a score ≥ 6 (90^th^ percentile).

Grade repetition was assessed with the question ‘Have you repeated school year(s) at primary school and middle school?’ (Never, at primary school, at middle school); multiple responses were possible. Grade repetition was defined as repeating at least one school-year. Last-trimester-school performance was assessed with the question ‘What is your average school-mark for the last-trimester?’ (≤7/20, 8-9/20, 10-13/20, 14-15/20, ≥16/20). Low school performance was defined as average school-mark below 10/20.

Cumulating mental health, behavioral and school difficulties (CD) was defined as the number of these outcome variables: alcohol use, tobacco use, cannabis use, hard drugs use, obesity, depressive symptoms, suicide attempts, involvement in violence, and low school performance (range 0 to 9). Grade repetition was not considered as it was close enough to low school performance. These variables were one-dimensional (as indicated by a factor analysis that yielded a first eigenvalue of 1.95 that was much higher than the 2^nd^ eigenvalue of 0.25) and their Cronbach's alpha was satisfactory (0.62) allowing the single score to be calculated. The CD was then categorized into: 0, 1, 2–3, and ≥4 (90^th^ percentile value).

### Statistical analysis

The mental health, behavioral and school difficulties studied were consumptions of alcohol, tobacco, cannabis, and hard drugs, obesity, depressive symptoms, suicide attempts, involvement in violence, grade repetition, and low school performance. The socioeconomic factors investigated were: gender, age, nationality, family structure, parents’ education, father’ occupation, and income. To assess the associations of mental health, behavioral and school difficulties with socioeconomic factors were used logistic regression models to compute gender-age-adjusted odds ratios (ORga) and odds ratios adjusted for all socioeconomic factors (OR_full model_), and 95% confidence intervals (CI). We also used ORga and OR_full model_ but computed by polynomial logistic models to examine the associations between cumulating mental health, behavioral and school difficulties (CD) and socioeconomic factors. The analyses were performed using the Stata program (Texas: Stata Corporation 2007).

## Results

The characteristics of subjects are shown in Table 2. Boys represented 49.9%. Mean age was 13.5 (SD 1.3) years. European and non-European immigrants represented respectively 3.5% and 3.5% of subjects. One quarter of adolescents lived with divorced/separated parents or in reconstructed families, 11.9% with single parents or other non-intact families. Half of subjects had low parents’ education, 32.5% low social status (manual workers 25.0% and inactive 7.5%), and 17.7% insufficient income. Alcohol use affected 35.2% of subjects, tobacco use 11.2%, cannabis use 5.6%, hard drugs use 2.8%, and obesity 10.6%. Depressive symptoms affected 13.3% of subjects, suicide attempts 9.9%, involvement in violence 10.3%, grade repetition 14.7%, and low school performance 8.2%. Multi-morbidity was very common: 44.1% for CD0, 30.8% for CD1, 18.4% for CD2-3, and 6.7% for CD ≥ 4.

**Table 2:** Characteristics of subjects (N = 1,559)

	**% or mean (SD)**
**Boys**	49.9 (1.3)
**Age (yr)**	
12 or under	38.7 (1.2)
13	23.9 (1.1)
14 or over	37.4 (1.2)
Mean	13.5 (1.3)
**Nationality**	
French	93.1 (0.6)
European immigrants	3.5 (0.5)
Non-European immigrants	3.5 (0.5)
**Family structure**	
Intact	63.0 (1.2)
Divorced/separated parents and reconstructed family	25.1 (1.1)
Single parent and others	11.9 (0.8)
**Low parents’ education**	48.7 (1.3)
**Father’s occupation**	
Managers, professionals, and intermediate professionals	38.2 (1.2)
Craftsmen, tradesmen, and firm heads	20.1 (1.0)
Service workers and clerks	9.2 (0.7)
Manual workers and other occupations	25.0 (1.1)
Inactive people	7.5 (0.7)
**Insufficient income**	17.7 (1.0)
**Mental health, behavioral, and school difficulties**	
*Last-30-day substance use*	
Alcohol	35.2 (1.2)
Tobacco	11.2 (0.8)
Cannabis	5.6 (0.6)
Hard drugs	2.8 (0.4)
Obesity	10.6 (0.8)
Depressive symptoms	13.3 (0.9)
Suicide attempts	9.9 (0.8)
Involvement in violence	10.3 (0.8)
Grade repetition	14.7 (0.9)
Low school performance (<10/20)	8.2 (0.7)
**Multi-morbidity**	
CD 0	44.1 (1.3)
CD 1	30.8 (1.2)
CD 2-3	18.4 (1.0)
CD ≥ 4	6.7 (0.6)

CD: number of these outcome variables alcohol use, tobacco use, cannabis use, hard drugs use, obesity, depressive symptoms, suicide attempts, involvement in violence, and low school performance (range 0 to 9).

Table 3 shows that boys had a higher risk for alcohol use, obesity, and involvement in violence (gender-age-adjusted odds ratios ORga 1.27, 1.53, and 2.51, respectively) but a lower risk for depressive symptoms and suicide attempts (ORga 0.35 and 0.54, respectively). The risk strongly increased with age (ORga reaching 5.51) except for hard drugs use, obesity, and suicide attempts. Compared with French adolescents, European immigrants had a higher risk for hard drugs use, suicide attempts, and grade repetition (ORga 2.06-3.60) while non-European immigrants had a higher risk for tobacco use, cannabis use, hard drugs use, involvement in violence, grade repetition, and low school performance (ORga 2.18-5.13). Compared with intact families’ adolescents, those with divorced/separated parents or reconstructed families had a higher risk for all substances uses, depressive symptoms, suicide attempts, involvement in violence, grade repetition, and low school performance (ORga 1.66-3.54); while those with single parents or other non-intact families had a higher risk for all outcomes variables except obesity and depressive symptoms (ORga 1.41-3.94). Low parents’ education played a protective role for alcohol use (ORga 0.74) but was a risk factor for obesity, depressive symptoms, suicide attempts, grade repetition, and low school performance (ORga 1.42-5.24). Insufficient income was associated with all outcome variables except alcohol use, cannabis use, and obesity (ORga 1.81-2.55). Compared with manager/professional/intermediate professional offspring, inactive offspring had a lower risk for alcohol use (ORga 0.64) but a higher risk for tobacco use, suicide attempts, and involvement in violence (ORga 1.97-2.77); manual-worker offspring had a higher risk for obesity (ORga 2.24); craftsman/tradesman/firm head offspring had a higher risk for tobacco use (ORga 1.89). Regarding low school performance a clear social gradient was revealed with ORga varying from 2.68 for craftsman/tradesman/firm head offspring to 6.97 for inactive offspring. Similar trend was found for grade repetition.

**Table 3:** Associations of mental health, behavioral, and school difficulties with socioeconomic factors among adolescents (N = 1559): gender-age-adjusted odds ratios and 95% confidence intervals

	**N**	**Alcohol use**	**Tobacco use**	**Cannabis use**	**Hard drugs use**	**Obesity**	**Depressive symptoms**	**Suicide attempts**	**Involvement in violence**	**Grade repetition**	**Low school-performance**
**Boys**	778	1.27* 1.03-1.58	0.82 0.60-1.13	1.55 0.99-2.42	1.45 0.78-2.68	**1.53**† 1.10-2.13	**0.35**‡ 0.26-0.49	**0.54**‡ 0.39-0.77	**2.51**‡ 1.77-3.57	1.12 0.84-1.49	0.97 0.67-1.39
**Age**											
12 or under	603	1.00	1.00	1.00	1.00	1.00	1.00	1.00	1.00	1.00	1.00
13	373	**2.09**‡ 1.57-2.78	0.93 0.56-1.55	1.32 0.61-2.86	1.66 0.71-3.86	0.86 0.57-1.29	1.36 0.90-2.05	0.85 0.54-1.36	1.16 0.73-1.84	**2.41**‡ 1.52-3.80	1.61 0.96-2.71
14 or over	583	**3.43**‡ 2.66-4.42	**2.87**‡ 1.97-4.17	**4.61**‡ 2.59-8.23	2.05 0.98-4.29	0.69 0.47-1.00	**1.85**‡ 1.30-2.62	1.30 0.89-1.89	**1.95**‡ 1.33-2.85	**5.51**‡ 3.73-8.13	**2.39**† 1.54-3.72
**Nationality**											
French	1,451	1.00	1.00	1.00	1.00	1.00	1.00	1.00	1.00	1.00	1.00
European immigrants	54	0.86 0.47-1.57	1.93 0.91-4.12	2.34 0.88-6.21	3.60* 1.22-10.6	0.67 0.24-1.88	1.45 0.70-2.99	**2.27**† 1.11-4.66	1.55 0.68-3.54	2.06* 1.04-4.08	1.26 0.49-3.25
Non-European immigrants	54	0.66 0.36-1.22	**2.41**† 1.24-4.68	2.75* 1.22-6.19	**5.13**‡ 2.03-12.9	1.29 0.57-2.92	1.10 0.50-2.42	1.90 0.90-3.99	2.18* 1.08-4.39	**3.49**‡ 1.90-6.40	**3.24**‡ 1.64-6.38
**Family structure**											
Intact	982	1.00	1.00	1.00	1.00	1.00	1.00	1.00	1.00	1.00	1.00
Divorced/separated parents and reconstructed family	391	**1.66**‡ 1.30-2.14	**3.54**‡ 2.47-5.06	**2.19**† 1.32-3.65	2.08* 1.02-4.27	0.93 0.63-1.38	**2.16**‡ 1.56-3.00	**2.54**‡ 1.75-3.69	1.92‡ 1.31-2.81	**2.23**‡ 1.61-3.10	**3.11**‡ 2.06-4.71
Single parent and others	186	1.41* 1.01-1.98	**2.59**‡ 1.62-4.16	**3.37**‡ 1.89-5.99	**3.94**‡ 1.84-8.41	1.21 0.74-1.96	1.00 0.60-1.66	**2.41**‡ 1.50-3.89	3.10‡ 1.99-4.84	**2.65**‡ 1.76-3.99	**3.50**‡ 2.12-5.77
**Low parents’ education**	759	**0.74**† 0.60-0.92	1.21 0.88-1.67	1.08 0.70-1.68	1.48 0.80-2.75	1.42* 1.02-1.97	1.46* 1.08-1.97	1.47* 1.05-2.07	1.30 0.93-1.81	**3.30**‡ 2.41-4.53	**5.24**‡ 3.32-8.29
**Father’ occupation**											
Managers, professionals, and intermediate professionals	595	1.00	1.00	1.00	1.00	1.00	1.00	1.00	1.00	1.00	1.00
Craftsmen, tradesmen, and firm heads	314	1.18 0.88-1.58	**1.89**† 1.22-2.94	1.66 0.96-2.89	1.64 0.78-3.46	0.73 0.43-1.26	1.09 0.72-1.65	1.54 0.96-2.45	1.23 0.78-1.93	1.75* 1.09-2.80	**2.68**† 1.40-5.14
Service workers and clerks	144	0.90 0.61-1.34	0.82 0.40-1.67	0.36 0.11-1.20	0.25 0.03-1.87	1.42 0.79-2.55	1.05 0.60-1.84	1.38 0.73-2.61	0.90 0.48-1.71	**2.11**† 1.20-3.73	**2.80**† 1.28-6.13
Manual workers and other occupations	389	0.92 0.70-1.22	1.50 0.98-2.28	0.74 0.40-1.36	0.85 0.37-1.94	**2.24**‡ 1.51-3.34	1.17 0.80-1.70	1.46 0.94-2.27	0.86 0.54-1.36	**3.62**‡ 2.44-5.37	**5.72**‡ 3.27-10.0
Inactive people	117	0.64* 0.41-1.00	2.00* 1.13-3.54	1.43 0.67-3.04	1.22 0.40-3.73	1.73 0.93-3.21	0.83 0.45-1.55	1.97* 1.08-3.60	**2.77**‡ 1.63-4.71	**5.79**‡ 3.49-9.63	**6.97**‡ 3.53-13.7
**Insufficient income**	276	1.10 0.83-1.45	**1.84**‡ 1.27-2.67	1.59 0.96-2.66	**2.55**† 1.34-4.85	1.38 0.93-2.05	**2.01**‡ 1.43-2.83	**2.48**‡ 1.71-3.58	**2.10**‡ 1.44-3.07	**1.84**‡ 1.31-2.58	**1.81**† 1.19-2.74

*p < 0.05, †p < 0.01, ‡p < 0.001.

The 95% confidence intervals are not provided to alleviate the Table.

N: number of subjects.

Table 4 displays odds ratios adjusted for all socioeconomic factors (full logistic models). European immigrants had a significant OR for hard drugs use only (3.68); non-European immigrants had significant ORs for cannabis use, hard drugs use, grade repetition, and low school performance (2.31-4.14). Having divorced/separated parents or reconstructed family had significant ORs for alcohol use, tobacco use, cannabis use, depressive symptoms, suicide attempts, involvement in violence, grade repetition, and low school performance (1.80-3.37); and having single parent or other type of non-intact family for all substances uses, suicide attempts, involvement in violence, grade repetition, and low school performance (1.64-3.72). Low parents’ education had significant ORs for alcohol use (0.71), depressive symptoms (1.42), grade repetition (2.15), and low school performance (3.32). Craftsman/tradesman/firm head offspring had a significant OR for tobacco use only (1.64); manual worker offspring for obesity (2.17), grade repetition (2.20), and low school performance (3.05); and inactive offspring for alcohol use (0.57), grade repetition (2.71), and low school performance (2.56). Insufficient income had significant ORs for tobacco use, hard drugs use, depressive symptoms, suicide attempts, and involvement in violence (1.51-2.33).

**Table 4:** Associations of mental health, behavioral, and school difficulties with socioeconomic factors (N = 1559): odds ratios adjusted for all socioeconomic factors and 95% confidence intervals

	**N**	**Alcohol use**	**Tobacco use**	**Cannabis use**	**Hard drugs use**	**Obesity**	**Depressive symptoms**	**Suicide attempts**	**Involvement in violence**	**Grade repetition**	**Low school-performance**
**Boys**	778	1.28* 1.03-1.60	0.85 0.61-1.18	1.63* 1.03-2.58	1.54 0.82-2.90	**1.56**† 1.12-2.18	**0.34**‡ 0.225-0.47	**0.55**‡ 0.39-0.78	**2.70**‡ 1.88-3.87	1.18 0.87-1.60	0.98 0.67-1.44
**Age**											
12 or under	603	1.00	1.00	1.00	1.00	1.00	1.00	1.00	1.00	1.00	1.00
13	373	**2.14**‡ 1.60-2.86	0.93 0.55-1.57	1.34 0.62-2.93	1.66 0.70-3.95	0.86 0.57-1.30	1.35 0.89-2.05	0.82 0.51-1.33	1.14 0.71-1.83	**2.48**‡ 1.55-3.97	1.67 0.97-2.87
14 or over	583	**3.63**‡ 2.80-4.70	**2.95**‡ 2.00-4.35	**4.95**‡ 2.74-8.96	2.07 0.97-4.45	0.63 0.43-0.92	**1.90**‡ 1.33-2.72	1.26 0.85-1.85	**1.91**‡ 1.29-2.82	**5.46**‡ 3.65-8.17	**2.14**‡ 1.34-3.39
**Nationality**											
French	1,451	1.00	1.00	1.00	1.00	1.00	1.00	1.00	1.00	1.00	1.00
European immigrants	54	0.87 0.47-1.61	1.68 0.76-3.68	2.43 0.89-6.62	3.68* 1.20-11.3	0.59 0.21-1.68	1.23 0.58-2.60	1.98 0.94-4.17	1.50 0.65-3.48	1.59 0.78-3.24	0.85 0.32-2.27
Non-European immigrants	54	0.64 0.35-1.22	1.99 0.99-4.01	2.35* 1.00-5.53	**4.14**† 1.54-11.2	1.17 0.51-2.70	1.01 0.45-2.27	1.47 0.68-3.17	1.62 0.77-3.37	**2.51**† 1.30-4.85	2.31* 1.11-4.81
**Family structure**											
Intact	982	1.00	1.00	1.00	1.00	1.00	1.00	1.00	1.00	1.00	1.00
Divorced/separated parents and reconstructed family	391	**1.81**‡ 1.40-2.34	**3.37**‡ 2.33-4.87	**2.24**† 1.33-3.77	1.94 0.93-4.05	0.83 0.56-1.24	**2.07**‡ 1.48-2.89	**2.27**‡ 1.55-3.33	**1.80**† 1.21-2.65	**1.77**‡ 1.26-2.50	**2.52**‡ 1.64-3.87
Single parent and others	186	**1.77**† 1.24-2.54	**2.31**‡ 1.39-3.84	**3.35**‡ 1.78-6.27	**3.72**† 1.63-8.50	1.03 0.61-1.73	0.96 0.56-1.64	**2.07**† 1.23-3.46	**2.43**‡ 1.49-3.94	1.64* 1.04-2.61	**2.23**† 1.28-3.86
**Low parents’ education**	759	**0.71**† 0.56-0.91	0.92 0.63-1.32	1.00 0.60-1.65	1.33 0.66-2.68	1.09 0.76-1.56	1.42* 1.02-1.98	1.16 0.80-1.69	1.08 0.74-1.58	**2.15**‡ 1.51-3.05	**3.32**‡ 2.03-5.42
**Father’ occupation**											
Managers, professionals, and intermediate professionals	595	1.00	1.00	1.00	1.00	1.00	1.00	1.00	1.00	1.00	1.00
Craftsmen, tradesmen, and firm heads	314	1.20 0.88-1.62	1.64* 1.04-2.59	1.36 0.76-2.43	1.17 0.53-2.57	0.73 0.42-1.25	0.93 0.61-1.43	1.28 0.79-2.08	1.06 0.66-1.69	1.35 0.83-2.20	1.96* 1.00-3.82
Service workers and clerks	144	0.92 0.62-1.39	0.64 0.31-1.36	0.28* 0.08-0.96	0.15 0.02-1.20	1.38 0.76-2.50	0.80 0.44-1.43	1.07 0.56-2.07	0.75 0.39-1.45	1.55 0.86-2.80	1.83 0.81-4.13
Manual workers and other occupations	389	0.97 0.72-1.32	1.14 0.71-1.83	0.53 0.27-1.06	0.45 0.18-1.15	**2.17**‡ 1.41-3.35	0.79 0.52-1.20	0.98 0.60-1.60	0.64 0.38-1.06	**2.20**‡ 1.43-3.40	**3.05**‡ 1.67-5.54
Inactive people	117	0.57* 0.35-0.93	1.13 0.59-2.17	0.65 0.27-1.56	0.36 0.10-1.29	1.60 0.81-3.16	0.52 0.26-1.03	1.00 0.51-1.97	1.48 0.80-2.71	**2.71**‡ 1.53-4.83	2.56* 1.21-5.42
**Insufficient income**	276	1.16 0.86-1.55	1.51* 1.02-2.26	1.47 0.85-2.56	2.33* 1.16-4.68	1.16 0.77-1.76	**1.90**‡ 1.32-2.73	**2.04**‡ 1.38-3.02	**1.84**† 1.23-2.76	1.12 0.78-1.62	1.02 0.65-1.59

*p < 0.05, †p < 0.01, ‡p < 0.001.

The 95% confidence intervals are not provided to alleviate the table.

N: number of subjects.

Note: Using stepwise forward procedure retaining only significant factors very little change the adjusted odds ratios.

Table 5 reveals the associations between cumulating mental health, behavioral, and school difficulties (CD) and socioeconomic factors. We found that all socioeconomic factors had non-significant adjusted odds ratios for CD1 (except age groups). But we observed impressive roles with strong adjusted odds ratio gradients from CD 2–3 to CD ≥ 4 for having divorced/separated parents or reconstructed families (ORs 2.31 and 4.86, respectively), single parents (ORs 2.32 and 3.78, respectively), and insufficient income (ORs 1.54 and 2.56, respectively). Being European immigrant or non-European one, low parents’ education, and lower fathers’ occupations had significant ORga for CD2-3 and/or CD ≥ 4, but they became non-significant when adjusting for all socioeconomic factors. Their associations with CD were thus entirely explained by family structure and insufficient income. Advancing age had higher risk for multi-morbidity which did not change when controlling for all socioeconomic factors.

**Table 5:** **Associations between cumulating mental health, behavioral, and school difficulties (CD)**^**a) **^**and socioeconomic factors: odds ratios and 95% confidence intervals**

		**Gender-age adjusted odds ratios (vs. CD0)**			**Odds ratios adjusted for all socioeconomic factors (vs. CD0)**		
	**N**	**CD1**	**CD2-3**	**CD ≥4**	**CD1**	**CD2-3**	**CD ≥4**
*Prevalence (%)*	*1,559*	*30.8*	*18.4*	*6.7*	*30.8*	*18.4*	*6.7*
**Boys**	778	1.20 0.94-1.52	1.11 0.84-1.47	1.09 0.72-1.65	1.20 0.95-1.52	1.11 0.84-1.48	1.18 0.76-1.83
**Age**							
12 or under	603	1.00	1.00	1.00	1.00	1.00	1.00
13	373	**1.67**‡ 1.24-2.26	**1.70**† 1.16-2.48	1.67 0.90-3.10	**1.70**‡ 1.26-2.31	**1.74**† 1.18-2.55	1.70 0.90-3.22
14 or over	583	**2.39**‡ 1.82-3.16	**3.45**‡ 2.48-4.79	**4.46**‡ 2.70-7.36	**2.40**‡ 1.82-3.18	**3.58**‡ 2.56-5.01	**4.77**‡ 2.82-8.07
**Nationality**							
French	1,451	1.00	1.00	1.00	1.00	1.00	1.00
European immigrants	54	0.55 0.26-1.17	0.88 0.40-1.93	2.82* 1.25-6.35	0.52 0.24-1.09	0.80 0.36-1.77	2.28 0.96-5.40
Non-European immigrants	54	0.94 0.48-1.87	1.02 0.46-2.22	2.41* 1.01-5.74	0.92 0.46-1.83	0.88 0.39-1.99	1.69 0.67-4.27
**Family structure**							
Intact	982	1.00	1.00	1.00	1.00	1.00	1.00
Divorced/separated parents and reconstructed family	391	1.25 0.94-1.68	**2.40**‡ 1.73-3.32	**5.50**‡ 3.37-8.96	1.22 0.90-1.64	**2.31**‡ 1.66-3.22	**4.86**‡ 2.95-8.02
Single parent and others	186	1.18 0.80-1.75	**2.16**‡ 1.41-3.33	**4.81**‡ 2.62-8.82	1.20 0.79-1.81	2.32* 1.47-3.67	**3.78**‡ 1.95-7.31
**Low parents’ education**	759	1.06 0.84-1.34	1.34* 1.01-1.77	**1.76**† 1.15-2.69	0.96 0.74-1.25	1.13 0.83-1.55	1.17 0.72-1.90
**Father’ occupation**							
Managers, professionals, and intermediate professionals	595	1.00	1.00	1.00	1.00	1.00	1.00
Craftsmen, tradesmen, and firm heads	314	1.21 0.88-1.67	1.07 0.71-1.60	**2.18**† 1.23-3.87	1.21 0.88-1.69	0.97 0.65-1.47	1.66 0.91-3.03
Service workers and clerks	144	1.14 0.74-1.76	1.47 0.90-2.39	0.49 0.14-1.66	1.12 0.72-1.73	1.27 0.76-2.10	0.32 0.09-1.14
Manual workers and other occupations	389	1.37* 1.01-1.87	1.54* 1.08-2.21	1.92* 1.09-3.37	1.35 0.97-1.88	1.21 0.82-1.80	1.09 0.58-2.06
Inactive people	117	1.04 0.64-1.70	1.00 0.56-1.80	**3.19**‡ 1.60-6.35	0.95 0.56-1.62	0.58 0.30-1.10	1.17 0.53-2.59
**Insufficient income**	276	1.35 0.98-1.87	**1.79**† 1.25-2.57	**3.38**‡ 2.12-5.40	1.29 0.92-1.80	1.54* 1.05-2.24	**2.56**‡ 1.55-4.23

*p < 0.05, †p < 0.01, ‡p < 0.001.

N: number of subjects.

a) CD was defined as the number of these outcome variable alcohol use, tobacco use, cannabis use, hard drugs use, obesity, depressive symptoms, suicide attempts, involvement in violence, and low school performance (range 0 to 9).

## Discussion

The present study is an original investigation assessing the concurrent roles of a wide range of socioeconomic factors in multi-morbidity defined as cumulating a number of mental health, behavioral, and school difficulties that are well known to be very common in early adolescence. We focused on early adolescence because multi-morbidity may be common and it should be evaluated, treated and monitored, especially among most vulnerable adolescents to prevent their aggravation in later adolescence [32,33].

Our study shows that many adolescents suffered from a wide range of mental health, behavioral, and school difficulties which resulted in very common multi-morbidity with 18.4% for CD2-3 and 6.7% for CD ≥ 4. This research reveals that multi-morbidity affected mainly most vulnerable adolescents with altered family functioning, deteriorated family environment, and insufficient income. Divorced/separated parents and reconstructed families appeared to be an important cause, maybe especially because they may result in lower material, financial, and mental resources, social supports, home change, and living environment change. Living with single parents was also a potential risk factor. One study reported that among middle school adolescents, parents’ divorce/separation and death early occurred (median ages of occurring 6 and 8 years, respectively) [18], mostly when the children are in primary school leading them to enter school/middle school with a variety of behavioral and emotional issues and lack of motivation for learning. These issues may be aggravated over time leading many adolescents to be early affected by several mental health, behavioral and school difficulties (median age of occurring 10 years for depressive symptoms; 11 years for suicide attempts and alcohol use initiation; 12 years for tobacco use initiation; and 13 years for cannabis and hard drugs uses initiation) [18]. Consequently, multi-morbidity may be early generated among adolescents. Another important result of our study is that being immigrant, low parents’ education, and low fathers’ occupations had significant gender-age-adjusted odds ratios for CD ≥ 2, but these became non-significant when adjusted for all socioeconomic factors. Non-intact families and insufficient income explained thus the higher risks associated with being immigrant, low parents’ education, and low father’s occupations. Families are the cornerstone of society and the main social environment for children. But families are changing leaving many families now live in non-traditional arrangements, and there are more cohabitations, divorces, and remarriages [19]. Over the past decade poverty in households with children is rising in nearly all OECD countries [19]. Our findings are consistent with two studies which showed that multi-morbidity also affects youth [48,49] and with studies in adults and elderly people that also point out the roles of socioeconomic deprivation, family structure, and income [40-47].

It should be noted that mental disorders of adolescents with poor social/material resources are less likely to be treated [55-58]. The mental disorders may thus increase over time. In France, people under poverty threshold (<60% of median income) represented 7.5% in 2009; it reached 13.7% in individual aged 18–24, 20.8% in single parent families, and 43.8% in inactive mothers [59]. Four million individuals had not complementary health insurance in 2008 [60]. Such issue also occurs in other countries, for example in the United States: in 2010, there were 46.2 million people in poverty and 49.9 million people without health insurance coverage (after a regular increase in the rate since 1999) [61]. Our findings point out that prevention to limit multi-morbidity should early identify children at risk, since they are in primary school and middle school, and to detect their difficulties which need to be treated and monitored over time.

Our study reveals that immigrants had a higher risk for CD ≥ 4 only and that the excess multi-morbidity mainly resulted from tobacco, cannabis, and hard drugs uses, suicide attempts, and school difficulty. This is expected as the problem may be exacerbated among immigrant adolescents because of lower parents’ education, socioeconomic status, and resources. We found that low parents’ education had a higher risk for both CD2-3 and CD ≥ 4, which mainly resulted from obesity, depressive symptoms, suicide attempts, and school difficulty. This knowledge of the nature of excess difficulties for immigrants and individuals with low parents’ education may be useful for promoting specific prevention.

Our results concerning social inequalities in multi-morbidity deserve particular interests. We found that manual-worker and inactive offspring had a strongly higher risk and it was explained by non-intact families and insufficient income. This finding highlights that studying social inequalities in multi-morbidity needs to take them into account in order to understand the mechanisms through which low parents’ occupations may favor multi-morbidity. Furthermore, our study shows that excess multi-morbidity for manual-worker and inactive offspring had as components tobacco use, obesity, suicide attempts, involvement in violence, and school difficulty. It should be noted that social inequalities observed in grade repetition and low school performance followed a strong gradient with gender-age-adjusted odds ratios up to 5.79 and 6.97, respectively.

The present study states that, compared with girls, boys had not a higher risk for multi-morbidity. This similarity concealed the higher risk for alcohol use, cannabis use, obesity, and involvement in violence, which was compensated by the lower risk for depressive symptoms and suicide attempts. Increasing age was associated with a higher risk for all CD1, CD2-3, and CD ≥ 4, which were attributed to alcohol use, tobacco use, cannabis use, depressive symptoms, and school difficulty. Age somewhat represents the duration of exposure, but also the latency time for mental health, behavioral, and school difficulties especially since the occurring of family issues. It is thus important to prevent these difficulties since an early age in order to avoid their cumulating over time. It may be noted that the gender and age differences did not change when controlling for all socioeconomic factors. These results are very troubling because they evidenced that mental health, behavioral, and school difficulties were not solved but cumulated and increased over time independently of socioeconomic factors. Each mental health, behavioral, or school difficulty appeared thus to be a motor for other difficulties. Several explanations may be advanced: (1) school and mental health difficulties are rather generally not solved but become more common and more persistent with time as a result the subjects affected may increase drugs uses to cope; (2) mental disorders such as depression are well known to affect executive functions, cognitive ability, and work performance [25-27] and may consequently aggravate adolescent situations; (3) alcohol, tobacco, and cannabis uses are well known to alter physical and mental capacities [34,35,37] leading to higher school and mental difficulties and higher drugs uses to cope.

### Strengths and limitations

The present study drew on a rich data set that allowed examination of the parts played by a number of socioeconomic factors in multi-morbidity in early adolescence. The participation rate was high (94%). Various measures have been used elsewhere [2,6,25,51,52,54]. The prevalence of health/behavior outcomes was similar with that of French adolescents. All were made to guarantee students’ anonymity. For this purpose birthday, birth place, residential town, school name, and precise class were not gathered. This study was based on self-reported data which are widely used to study adolescent living conditions, mental health, and unhealthy behaviors [2,4,32]. The obesity BMI threshold values used which are recommended by the National Institute for Health and Medical Research for studies in French adolescents, are appropriate and lower than international standards such as those recommended by the World Health Organization and the International Obesity Taskforce [53,62,63]. The socioeconomic issues generally occur before mental health, behavioral, and school difficulties, especially multi-morbidity as cumulating several of these difficulties. However causal relationships cannot be drawn leaving finding interpretation to be cautious. Certain factors such as genetic and personality features were not investigated. Given the number of statistical tests carried out, type I error may be a concern, but most tests were significant at the 0.001 level, with very large OR estimates.

## Conclusion

Multi-morbidity was common and included a wide range of mental health, behavioral, and school difficulties in early adolescence. Living with divorced/separated parents, in reconstructed families, with single parents, and insufficient income played impressive roles. Therefore, multi-morbidity affected mainly most vulnerable adolescents in poor families and with altered family functioning or deteriorated family environment. Divorce and separation of parents appeared to be an important risk factor, maybe especially because they may result in lower material, financial, and mental resources, home change, and living environment change. Living with single parents was also a potential risk factor. Being immigrant, low parents’ education, and low fathers’ occupations also played strong roles but these were explained by non-intact families and insufficient income. Prevention against multi-morbidity should be designed to help adolescents to solve their difficulties since an early age, especially among adolescents with socioeconomic issues, and particularly with family dysfunctioning and financial issues.

## Competing interests

The authors declare that they have no conflict of interest.

## Authors’ contributions

KC conceived the survey, carried out the study and had the main responsibility for writing the manuscript. MB participated in conceiving the study and writing the manuscript. NC participated in conceiving the survey, statistical analyses and writing the manuscript. All authors read and approved the final manuscript.
